# Which Factors Influence the Need for Inpatient Aftercare of Elderly Patients After Hospital Treatment for Proximal Humerus Fractures?

**DOI:** 10.1177/21514593251325365

**Published:** 2025-03-18

**Authors:** Bastian Mester, Raed Maali, Heinz-Lothar Meyer, Christina Polan, Stephanie Herbstreit, Monika Herten, Lars Becker, Marcel Dudda, Manuel Burggraf

**Affiliations:** 1 Department of Trauma, Hand and Reconstructive Surgery, 39081 University Hospital Essen, Essen, Germany; 2 University Health Orthopaedics, 12273 University of Missouri, Kansas City, MO, USA; 3 Department of Orthopaedics and Trauma Surgery, BG-Klinikum Duisburg, University of Duisburg-Essen, Duisburg, Germany; 4 Department of Orthopaedics and Trauma Surgery, GFO Kliniken Mettmann-Süd, Langenfeld, Germany

**Keywords:** geriatrics, geriatric traumatology, proximal humerus fracture, influencing factors, need for care after inpatient treatment

## Abstract

**Introduction:**

While epidemiology and treatment strategies of proximal humerus fractures have been well studied, post-hospital care is poorly analysed. Corresponding data is available in the context of hip fractures, but the evidence regarding proximal humerus fractures is weak. Aim of this study is to identify risk factors for institutionalisation required after discharge into inpatient aftercare for elderly patients treated for proximal humerus fractures.

**Materials and Methods:**

For this retrospective single-centre investigation, n = 295 patients (age 70 (58,79) years, 63.7% female) admitted to hospital from home due to proximal humerus fractures were included and divided into two study groups: Patients being discharged home (*‘Home’*) vs being discharged into aftercare (*‘Aftercare’*). Differences regarding demographic and clinical data were analysed. Odds ratios (OR) of influencing factors (adjusted for age) were calculated by logistic regression analysis.

**Results:**

Increased age notably increased the likelihood for discharge of patients into ‘Aftercare’ (OR 1.09 [1.06;1.12] per year of life). Age-independent indicators for ‘Aftercare’ were higher ASA score (OR 2.16 per ASA point [1.37;3.49]; *P* < .001), anterior surgical approach (OR 6.05 [1.93,27.1]; *P* < .006), duration of surgery (OR 1.01 per min [1.00,1.02]; *P* < .012), non-surgical complications (OR 3.82 [1.60,9.49]; *P* < .003), length of stay (OR 1.12 per day [1.04,1.22]; *P* < .005), ICU stay (OR 3.15 [1.71,6.00]; *P* < .001) and reversely surgery (OR 0.39 [0.19,0.80]; *P* < .010).

**Conclusion:**

Increased Age and higher ASA score notably increase the likelihood for post-hospital discharge to an inpatient aftercare facility. Available literature in the context of hip fractures is confirmed. The results of this study may assist in identifying patients at risk and may serve as a stepstone in establishing a scoring system for elderly patients with proximal humerus fractures.

## Introduction

Proximal humerus fractures belong to the most common fracture entities.^1^ Recent studies report that the actual incidence of proximal humerus fractures is higher than previously assumed with 90.8 – 110.0 per 100,000 patients per year.^2,3^ These fractures are strongly associated with osteoporosis and generally considered a characteristic indicator fracture.^4^ Subsequently, it is the third leading fracture in the geriatric population following the proximal femur and distal radius, with an upward trend of incidence due to the demographic changes.^4–6^

There has been an enormous research effort in the past regarding epidemiologic factors, complications, and outcomes of conservative vs (vs) operative treatment of proximal humeral fractures. Different treatment modalities in the elderly population have been discussed controversially: Recent investigations indicate similar early outcomes of conservative treatment compared to surgery.^7,8^ Regarding the different surgical strategies available, a trend towards a more liberal application of primary reverse arthroplasty can be observed.^5^

In contrast to patients with proximal femur fractures, who frequently have been living under supervision or in a nursing home before, elderly patients with proximal humerus fractures are mostly admitted to hospital from a self-sustaining living situation, according to literature in 70 - 90% of admissions.^9,10^

Interdisciplinary orthogeriatric treatment concepts aim to retain patient’s activity and autonomy. Several studies investigating patients with proximal femoral fractures were able to proof positive effects with more favourable functional outcome parameters, reduced mortality rates and especially a lower risk for institutionalisation after discharge into inpatient aftercare with social dependency, e.g. geriatric wards or nursing homes.^11–14^ Though, this evidence is not available for proximal humeral fractures in the geriatric population. Finally, it remains unclear which patients are at risk for an inpatient aftercare setting with social dependency and presumably will benefit from orthogeriatric measures and early social service support.

The aim of this study is to characterize geriatric patients with inpatient treatment of proximal humeral fractures being discharged home in a self-sustaining living situation vs patients being discharged into an inpatient aftercare setting with social dependency. Furthermore, this investigation aims to identify patient- and treatment-associated risk factors for institutionalisation required after discharge into inpatient aftercare with social dependency.

## Materials and Methods

For this retrospective single-centre investigation, we identified all patients being hospitalized between 01/2015 and 12/2019 in a level-one trauma department due to proximal humeral fractures – either having been treated conservatively or operatively – by software-assisted screening of the local patient correspondence database. All patients have been hospitalized to provide sufficient analgesia and nursing in the early phase after proximal humerus fracture and – in cases selected for operation – for preparation and carrying out the surgery itself. The initially identified n = 454 patients were then further assessed for eligibility. The inpatient treatment records as well as the discharge letters were screened regarding the social history and living situation before and after hospitalization: patients who were admitted to hospital from a self-sustaining living situation at home were further analysed, patients who have been socially dependent or residing in a care / nursing home (as well as cases with unknown living situations) were excluded. Thus, an equal baseline independence level of all study participants can be assumed. Further exclusion criteria were multiple trauma, open fractures and pathologic fractures associated with primary/secondary bone cancer. Subsequently, n = 295 patients were finally included in this study.

Ethical approval was obtained from the local ethical committee (No. 20-9134-BO). The study was performed in accordance with the guidelines of the World Medical Association Declaration of Helsinki. The requirement for acquisition of informed consent from the patients was waived because of the retrospective nature of the study.

Regarding the living situation after hospitalization, the cohort was divided into two subgroups: patients being discharged home in a self-sustaining living situation (*‘Home’*) vs patients being discharged into an inpatient aftercare setting with social dependency including geriatric wards and nursing homes (*‘Aftercare’*).

Thus, inclusion criteria for study participants were inpatient treatment for proximal humerus fractures, conservative or operative treatment.

Exclusion criteria were social dependency before admission or unknown status, polytrauma, open or pathologic fractures.

**Figure 1.**
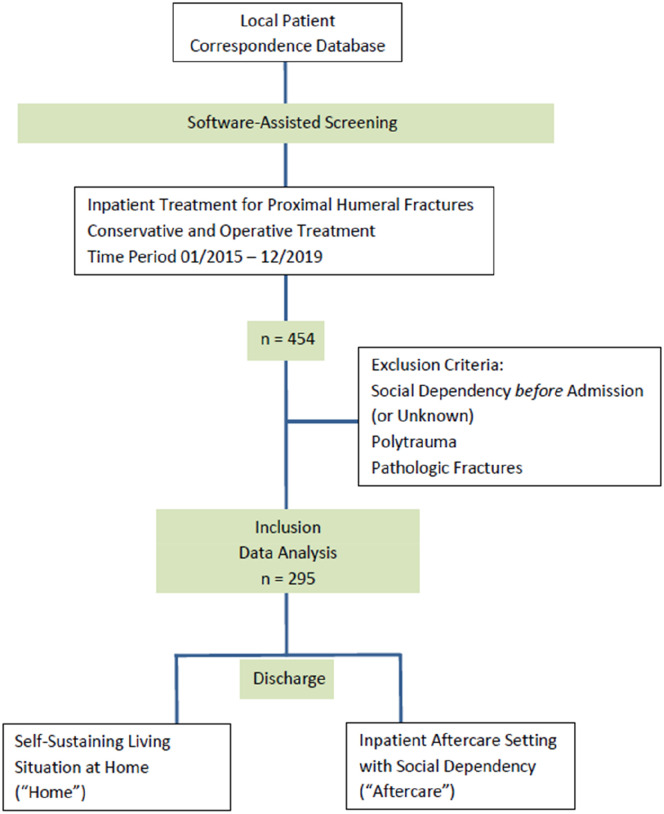
Flowchart presenting the enrolment of the final study population according to.^15^ Inclusion and data analysis of n = 295 patients; subgroups ‘Home’ vs ‘Aftercare’ depending on the living situation after discharge from hospital, treatment for proximal humeral fractures.

The patient’s enrolment process, application of inclusion and exclusion criteria and subgroup division is shown in Figure 1.^15^

Demographic, clinical as well radiological data in conjunction with the inpatient treatment were retrieved from the local patient database: age, gender, Body Mass Index (BMI), American Society of Anaesthesiologists (ASA) physical status classification system,^16^ pre-existing conditions, diabetes, malignancies, immunosuppression, smoking, substance abuse. Fracture classification according to Resch et al,^17^ operative vs conservative treatment, type of surgical treatment, surgical approach (anterior = deltopectoral approach), duration of surgery, non-surgical complications, length of stay, ICU (intensive care unit) stay.

In order to further classify the subgroups ‘Home’ and ‘Aftercare’ and to identify influencing factors, differences regarding demographic and clinical characteristics between the groups were analysed and logistic regression analysis was used to calculate odds ratios (OR) of possible influencing factors, adjusted for age.

### Statistical Analysis

IBM SPSS Version 27.0 and GraphPad Prism Version 9.4 were used to analyse the date and to compile the graphs. After performing a Shapiro-Wilk-Test, differences between the two groups were estimated by the Mann-Whitney *U* test in continuous and ordinal variables, whereas differences in categorical variables were assessed by a chi-square test (*X*^*2*^). Data are expressed as median and interquartile range (*Mdn* [IQR]) for continuous variables, ordinal and categorical variables are expressed as absolute numbers with percentage. The individual effect of each variable on the likelihood of ‘Aftercare’ was tested by logistic regression analysis. Results are reported as crude odds ratios (OR) and OR adjusted for age together with 95 % confidence interval (95% CI). As the analysis was strictly exploratory, no correction for multiple testing was performed.

In a second step, multiple logistic regression analysis was carried out to analyse how well different models of demographic variables predict ‘Aftercare’. Cases with missing variables were excluded. The predictive accuracy of these models was analysed by use of the Log-likelihood ratio test. To compare the model fit, the receiver operating characteristic (ROC) curve and the area under the ROC curve (AUC) were computed. Besides the percentage of patients classified correctly, positive predictive power (PPP) and negative predictive power (NPP) are given. To determine if a simpler model with less variables is (statistically) preferable, the likelihood ratio test (LRT) was performed. For all tests besides LRT, statistical significance was assumed for *P* < .05.

## Results

N = 295 patients admitted to hospital from a self-sustaining living situation at home were included. The median age was 70 (58, 79) years, the distribution female to male was 188 vs 107 patients. A proportion 52.2 % had presented with an ASA status ≥ 3, and 92.5% had at least one pre-existing medical condition (42.0% ≥ 4 conditions).

Regarding the type of the proximal humeral fracture according to Resch et al, 20.0% were classified type 1, 25.4% type 2, 16.9% type 3, 24.1% type 4 and 13.6 % type 5. N = 238 patients (80.7%) were treated operatively. Within this subgroup, the majority (77.4%) received open reduction and internal fixation (ORIF) via an anterior approach, 12.1% underwent primary shoulder arthroplasty.

Notable differences between the study groups were found for age, gender, ASA, pre-existing conditions, diabetes, smoking, surgery, anterior surgical approach, non-surgical complications, length of hospital stay and ICU stay, respectively (see Table 1). All demographic and clinical characteristics of the study population are summarized in Table 1.

**Table 1.** Patients Demographic and Clinical Characteristics.

Variable	Total	Home	Aftercare	*Z*/*X*^*2*^ (*φ*)	*P*
Age (years); N = 295	70 (58, 79)	65 (25, 75)	80 (75, 88)	7.79	<.001
Gender (female); N = 295	188 (63.7)	127 (59.9)	61 (73.5)	4.77 (0.13)	.029
BMI (kg/m^2^); N = 275	25.8 (22.6, 29.7)	26.0 (22.6, 30.4)	25.5 (22.5, 29.4)	1.17	.241
ASA; N = 295					
1	15 (5.1)	15 (7.1)	0 (−)	5.48	<.001
2	120 (40.7)	101 (47.6)	19 (22.9)		
3	136 (46.1)	86 (40.6)	50 (60.2)		
4	24 (8.1)	10 (4.7)	14 (16.9)		
Pre-existing conditions; N = 295 none	22 (7.5)	21 (9.9)	1 (1.2)	5.53	<.001
1 – 3	149 (50.5)	121 (57.1)	28 (33.7)		
4 – 5	62 (21.0)	40 (18.9)	22 (26.5)		
>5	62 (21.0)	30 (14.2)	32 (38.6)		
Diabetes (yes); N = 291	53 (18.2)	32 (15.4)	21 (25.3)	3.92 (−0.12)	.048
Malignancies (yes); N = 293	29 (9.9)	17 (8.1)	12 (14.6)	2.87 (−0.10)	.091
Immunosuppressed (yes); N = 292	6 (2.1)	4 (1.9)	2 (2.4)	0.07 (−0.02)	.788
Smoking (yes); N = 278	77 (27.7)	66 (33.2)	11 (13.9)	10.46 (0.19)	.001
Substance abuse (yes); N = 250	31 (12.4)	25 (14.5)	6 (7.7)	2.31 (0.10)	.128
Resch classification; N = 295					
1	59 (20.0)	44 (20.8)	15 (18.1)	0.48	.630
2	75 (25.4)	48 (22.6)	27 (32.5)		
3	50 (16.9)	38 (17.9)	12 (14.5)		
4	71 (24.1)	53 (25.0)	18 (21.7)		
5	40 (13.6)	29 (13.7)	11 (13.3)		
Surgery (yes); N = 295	238 (80.7)	184 (86.8)	54 (65.1)	18.07 (0.25)	<.001
Surgical treatment (arthroplasty); N = 232	30 (12.9)	19 (10.6)	11 (20.8)	3.74 (0.13)	.053
Surgical approach (anterior); N = 234	181 (77.4)	131 (72.4)	50 (94.3)	11.29 (0.22)	.001
Duration of surgery (min); N = 238	107 (86, 132)	105 (86, 132)	109 (86, 142)	0.81	.418
Non-surgical complications (yes); N = 295	30 (10.2)	12 (5.7)	18 (21.7)	16.77 (−0.24)	<.001
Length of stay (days); N = 295					
Surgery	6 (4, 8)	5 (3, 7)	8 (6, 10)	5.39	<.001
Conservative	6 (3, 12)	4 (2, 9)	8 (4, 13)	2.13	.033
ICU stay (yes); N = 295	164 (55.6)	103 (48.6)	61 (73.5)	14.99 (−0.23)	<.001

Data presented as Mdn [IQR] or absolute numbers and percentage of hundred related to the respective (sub)group (%). *N* total group size per variable, BMI body mass index, ASA American society of anesthesiologists physical status classification system, ICU intensive care unit. *Z* Mann-Whitney *U* test or *X*^*2*^ chi-square test statistics, *φ* phi coefficient. Deviations from the total population size result from the consideration of subgroups and missing values.

Logistic regression analysis on age revealed a notable increase in the likelihood for a post-hospital discharge to ‘Aftercare’, with a crude OR of 1.09 [1.06; 1.12] per additional year of life.

Adjusted for age, a higher patient’s ASA score was identified as an independent indicator for the ‘Aftercare’ group (OR 2.16 per ASA point [1.37; 3.49]; *P* < .001). The choice of an anterior surgical approach (OR 6.05 [1.93, 27.1]; *P* < .006), duration of surgery (OR 1.01 per min [1.00, 1.02]; *P* < .012), number of non-surgical complications (OR 3.82 [1.60, 9.49]; *P* < .003), total length of stay both for surgical (OR 1.12 per day [1.04, 1.22]; *P* < .005) and conservative treatment (OR 1.11 per day [1.02, 1.22]; *P* < .026) as well as an ICU stay (OR 3.15 [1.71, 6.00]; *P* < .001) were also age-independent risk factors for ‘Aftercare’. In contrary, surgical treatment was found to be a negative predictive factor for ‘Aftercare’ (OR 0.39 [0.19, 0.80]; *P* < .010).

The results of the logistic regression analysis (crude and adjusted for age) are presented in Table 2.

**Table 2.** Crude and Adjusted Odds Ratios of Potential Indicators for ‘Aftercare’.

Variable	Crude		Adjusted	
	*OR* [95% CI]	*P*	*OR* [95% CI]	*P*
Age (per year)	1.09 [1.06, 1.12]	<.001	N/A	N/A
Gender				
Female	1 (reference)		1 (reference)	
Male	0.54 [0.30, 0.93]	.030	1.09 [0.57, 2.09]	.792
BMI (kg/m^2^)	0.96 [0.92, 1.01]	.123	0.97 [0.91, 1.02]	.259
ASA	3.06 [2.04, 4.74]	<.001	2.16 [1.37, 3.49]	.001
Pre-existing conditions none	1 (reference)		1 (reference)	
1 – 3	4.86 [0.95, 88.9]	.130	1.92 [0.32, 37.3]	.555
4 – 5	11.5 [2.18, 214]	.021	4.06 [0.64, 80.1]	.211
>5	22.4 [4.27, 414]	.003	5.10 [0.80, 101]	.146
Diabetes (yes)	1.86 [0.99, 3.46]	.050	1.94 [0.97, 3.90]	.060
Malignancies (yes)	1.96 [0.87, 4.27]	.095	1.48 [0.60, 3.60]	.386
Immunosuppressed (yes)	1.27 [0.17, 6.61]	.788	2.50 [0.31, 15.5]	.337
Smoking (yes)	0.33 [0.15, 0.64]	.002	0.78 [0.34, 1.72]	.545
Substance abuse (yes)	0.49 [0.18, 1.18]	.135	1.34 [0.44, 3.76]	.592
Resch classification				
1	1 (reference)		1 (reference)	
2	1.65 [0.79, 3.56]	.192	1.41 [0.60, 3.40]	.434
3	0.93 [0.38, 2.22]	.864	0.72 [0.27, 1.89]	.503
4	1.00 [0.45, 2.22]	.993	0.83 [0.34, 2.05]	.686
5	1.11 [0.44, 2.75]	.818	1.36 [0.48, 3.84]	.554
Surgery (yes)	0.28 [0.16, 0.52]	<.001	0.39 [0.19, 0.80]	.010
Surgical treatment (arthroplasty)	2.21 [0.95, 4.93]	.058	1.63 [0.67, 3.82]	.264
Surgical approach (anterior)*	6.36 [2.20, 27.0]	.003	6.05 [1.93, 27.1]	.006
Duration of surgery (per min)	1.00 [1.00, 1.01]	.206	1.01 [1.00, 1.02]	.012
Non-surgical complications (yes)	4.62 [2.13, 10.3]	<.001	3.82 [1.60, 9.49]	.003
Length of stay (per day)				
Surgery	1.14 [1.06, 1.24]	.001	1.12 [1.04, 1.22]	.005
Conservative	1.06 [0.99, 1.15]	.101	1.11 [1.02, 1.22]	.026
ICU stay (yes)	2.93 [1.70, 5.21]	<.001	3.15 [1.71, 6.00]	<.001

Data of logistic regression analysis presented as crude odds ratio (*OR*) with 95% confidence interval (95% CI) as well as adjusted for age (*surgical approach adjusted for age and surgical treatment). BMI body mass index, ASA American society of anesthesiologists physical status classification system, ICU intensive care unit.

Multiple logistic regression tested the effect of a model comprising a set of patient demographics selected according to clinical experience (age, gender, BMI, malignancies, pre-existing conditions, ASA, diabetes, immunosuppression, smoking and substance abuse) on the likelihood of ‘Aftercare’ (model 1). This model was statistically significant when compared to the null hypothesis (Log-likelihood ratio 78.3 [*P* < .001]; AUC 0.837 [0.786, 0.888; *P* < .001]). Overall, 76.8% of the patients were classified correctly (NPP 80.9 %, PPP 63.6 %). When only using age and ASA (model 2), statistical significance was slightly lower (Log-likelihood ratio 67.8 [*P* < .001]; AUC 0.820 [0.765, 0.875; *P* < .001]) with 76.0 % of the patients identified correctly (NPP 79.0 %, PPP 63.8 %). Nevertheless, according to LRT, the (simpler) model 2 is the preferred model (10.5; *P* = .398).

**Figure 2.**
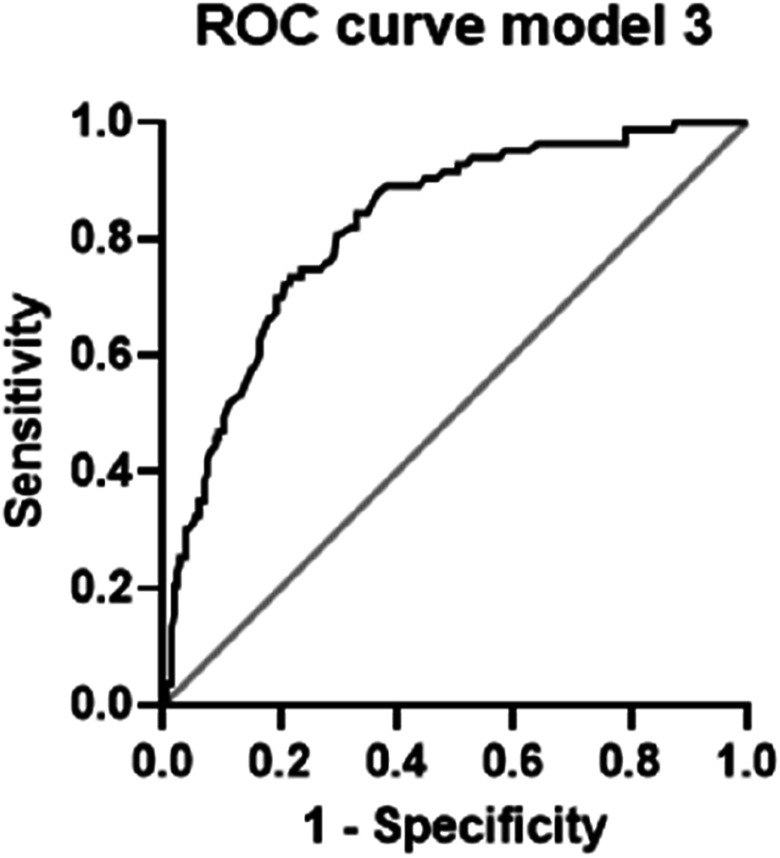
ROC curve of model 3 including age, ASA and non-operative treatment (AUC 0.823; *P* < .001). ROC receiver operating characteristic, ASA American society of anesthesiologists physical status classification system, AUC area under the ROC curve.

Finally, a combined model of age, ASA and non-operative treatment (model 3) yielded a Log-likelihood ratio of 81.8 (*P* < .001) and an AUC of 0.823 (0.772, 0.874; *P* < .001). The ROC curve of this model 3 is shown in Figure 2. By use of this model, 78.3 % of the patients were classified correctly with a NPP of 80.6 % and a PPP of 67.9 %. In comparison with model 2, model 3 is preferable according to LRT (5.40; *P* = .020).

Full regression results of model 2 and model 3 are provided in the supplement section (Supplements 1 and 2).

## Discussion

The main goal of this study was to depict demographic and clinical characteristics of a cohort of geriatric patients with inpatient treatment of proximal humeral fractures, comparing those being discharged in a self-sustaining living situation (‘Home’) vs patients with requirement for an inpatient aftercare setting (‘Aftercare’). Consequently, risk factors for institutionalisation after discharge (‘Aftercare’) were evaluated. As the most important findings, he main risk factor for the requirement of an inpatient aftercare was increased age. As age-independent risk factors, higher ASA score, non-operative treatment, duration of surgery, non-surgical complications, total duration of hospital stay and requirement for a temporary ICU stay could be identified.

Court-Brown et al. presented a 5-year prospective study of the epidemiology of 1027 proximal humeral fractures in 2009, focussing on epidemiological factors.^10^ Patients in this cohort were predominantly female (73%) with an average age of 66 (13, 98) years, were generally considered healthy, and nine-tenths lived at home in a self-sustaining living situation. Only 16% required home help. Age and gender characteristics in the present study were found to match to these data, investigating patients predominantly female in a slightly lower proportion (63.7%) and a similar average age of 70 (58, 79) years. The operation rate (80.7 %) is remarkably high in the recent study in comparison to the literature and is most likely attributed to the treatment philosophy of the study institution. On the other hand, demographic data of the present study are supported by the findings of several other investigations^1,5,9,18^ and can be regarded representative for proximal humeral fractures in the elderly population. It must be considered that the ratio of conservative vs operative treatment differs substantially between regions and study populations.^1,5,7,8,18,19^

In 2019, Adam et al. examined the influence of sustaining a proximal humeral fracture on mortality rates and identified variables predictive of five-year mortality on 288 patients.^20^ Comorbidities were detected in 81.6 % of the cohort. The authors identified a significant association between every measure of the patients’ physical and social independence and 5-year mortality. Comorbidities as well as employment status - which is regarded as one of the greatest measures of patient’s social independence - were found to be independent risk factors influencing the 5-year mortality rate. This has been confirmed by other authors in the past.^21–23^ Accordingly, in the present study a higher ASA score was an age-independent risk factor for loss of self-supply after discharge. In clinical practice, patients with higher ASA scores and multiple comorbidities are more likely subject to conservative treatment protocols because of specific surgical and anaesthesiologic risks. As these patients were also prone to the need for inpatient aftercare, surgery as a negative predictor for ‘Aftercare’ can be adequately explained in this context.

As there are no comparable studies available evaluating independent risk factors for institutionalisation required after discharge into inpatient aftercare due to proximal humeral fractures, the present study results may be set in relation to literature investigating the abovementioned risk factors in the setting of geriatric hip fractures.

In their prospective observational cohort study of 480 hip fracture patients, Moerman et al could show that only 24% of patients returned to their baseline instrumental activities of daily living.^24^ Factors associated with a larger loss of activities of daily living were increased age, pre-fracture living at home, pre-fracture use of walking aids and longer length of hospital stay. In contrast to this, a prospective observational study on health care situation and outcome of patients with femoral fracture by Endres et al, including 12,520 patients, found out that only 4.6% of femoral fracture patients experienced changes in their living situation post discharge.^25^ Though, over 50% of the cohort lost its ability to walk without assistive devices, being able to do so prior to the fracture event (65.1 %).

Van Dartel et al conducted a retrospective cohort study on geriatric patients after hospitalization for hip fracture surgery to investigate early predictors for discharge to a geriatric rehabilitation or nursing home vs discharge home.^26^ The purpose and study design were similar to the present study on geriatric patients with proximal humerus fractures. A total of 21,176 patients with hip fracture aged ≥70 years registered in the Dutch Hip Fracture Audit database were included. Matching the results of the present study, increased age and higher ASA score were – among others - identified as independent indicators for discharge to a geriatric ward or nursing home. Thereby, the results of the present study have been confirmed in the context of hip fractures before.^24–27^ This implicates that an equivalent risk profile for institutionalisation required after discharge into inpatient aftercare can be drawn for both hip fractures and proximal humerus fractures in the elderly population.

Two further studies evaluated the impact of certain scoring systems on the probability of adverse discharge disposition, synonymous with the need for assistance after discharge in a nursing home.^28,29^ According to their results, the ‘Adverse Discharge in Older Patients after Lower-extremity Surgery’ (ADELES) Risk Score as well as the ‘Identification of Seniors at Risk’ (ISAR) Score both have significant predictive power for discharge after hospital in the collective of geriatric lower extremity and hip trauma patients, respectively. The ADELES Risk Score is comprised of socioeconomic and surgical factors,^28^ the ISAR Score mainly consists of questions regarding social autonomy and illness level.^30^ According to the authors introducing the ADELES Risk Score, the predictive performance is characterized by an area under the ROC curve (AUC) of 0.85 (95% confidence interval; 0.84, 0.85), indicating excellent discriminative ability, which is typically assumed with an AUC > 0.80.^31^ In the present study - although not representing a score - a similar excellent result was demonstrated (ROC *model 3* including age, ASA, and non-operative treatment; AUC 0.823; *P* < .001) by use of only three easily and instantly available predictors. Therefore, it may serve as basis for further study efforts to establish a validated scoring system to estimate the risk for not being discharged home also for geriatric proximal humerus fractures.

Several concepts of orthogeriatric co-management for elderly trauma patients were proposed over the past 50 years, and evidence for substantial benefits has grown exponentially.^32^ Though, this evidence is primarily based on research related to proximal femoral fractures.

Wang et al published a meta-analysis of randomized controlled trials on the influence of inpatient comprehensive geriatric care on elderly patients with hip fractures,^13^ 15 trials with 3458 patients could be included. Amongst other positive effects in context with activities of daily life, it was shown that the proportion of patients who were discharged from hospital to the same place of residence as before the fracture was higher in the intervention group (comprehensive geriatric care) than in controls. Further advantages for patients with hip fractures regarding mortality, outcome and cost-effectiveness could also be demonstrated in the past.^11,12,14,33^ In contrast to this, evidence for orthogeriatric management concepts in elderly patients suffering from trauma of other body regions, e.g. proximal humeral fractures, is mainly not obtainable.^34^ Based on the data available regarding proximal hip fractures, it can be assumed that implementation of those orthogeriatric management concepts in the treatment of patients with proximal humerus fractures will most probably reduce the need for inpatient aftercare following hospital stay. Patients hospitalized due to proximal humerus fractures – both for conservative and surgical strategy - should undergo a geriatric assessment at the beginning.^35^ The results of the present study may therefore help to identify patients at risk for inpatient aftercare. As it is currently mandatory in the treatment of geriatric proximal femur fractures in Germany,^36^ those patients should be accordingly supervised by interdisciplinary multiprofessional teams, comprised of geriatricians, nurses, physiotherapists, social workers and trauma surgeons, in order to increase patient’s likelihood of being alive and in their own homes.^14,35,37,38^

### Limitations

The limitations of the study include the retrospective study design and the lack of a control group. A power analysis for sample size calculation was not performed. The number of available data for each parameter was included in the tables. Missing data handling by pairwise deletion may produce unbiased estimates. As is standard for multivariate analyses, cases with missing variables were excluded. The individual effect of each variable was tested by logistic regression analysis. OR were presented adjusted for age to eliminate confounding. Though, the remaining outcome variables may be interrelated, raining the possibility of confounding factors. An external validation or cross-validation of the logistic regression model is not available.

## Conclusion

With increasing age and higher ASA score of the patients with proximal humerus fractures as well as certain treatment-associated parameters, there is a notable increase in the likelihood for a post-hospital discharge to an inpatient aftercare facility. Overall, available literature in the context of hip fractures is confirmed. The results of this study may assist the practitioner in identifying patients at risk and streamline patient and family expectations. Furthermore, it may serve as a stepstone in establishing a scoring system for elderly patients with proximal humerus fractures to initiate a proper transfer to an aftercare facility early during the inpatient stay. Implementation of orthogeriatric management concepts may reduce the need for inpatient aftercare following hospital stay.

## Supplemental Material

**Supplemental Material -** Which Factors Influence the Need for Inpatient Aftercare of Elderly Patients After Hospital Treatment for Proximal Humerus Fractures? Supplemental Material for Which Factors Influence the Need for Inpatient Aftercare of Elderly Patients After Hospital Treatment for Proximal Humerus Fractures? by Bastian Mester, Raed Maali, Heinz-Lothar Meyer, Christina Polan, Stephanie Herbstreit, Monika Herten, Lars Becker, Marcel Dudda, and Manuel Burggraf in Geriatric Orthopaedic Surgery & Rehabilitation.

**Supplemental Material -** Which Factors Influence the Need for Inpatient Aftercare of Elderly Patients After Hospital Treatment for Proximal Humerus Fractures? Supplemental Material for Which Factors Influence the Need for Inpatient Aftercare of Elderly Patients After Hospital Treatment for Proximal Humerus Fractures? by Bastian Mester, Raed Maali, Heinz-Lothar Meyer, Christina Polan, Stephanie Herbstreit, Monika Herten, Lars Becker, Marcel Dudda, and Manuel Burggraf in Geriatric Orthopaedic Surgery & Rehabilitation.
